# The epidemiology of *Plasmodium falciparum* and *Plasmodium vivax* in East Sepik Province, Papua New Guinea, pre- and post-implementation of national malaria control efforts

**DOI:** 10.1186/s12936-020-03265-x

**Published:** 2020-06-05

**Authors:** Johanna H. Kattenberg, Dulcie L. Gumal, Maria Ome-Kaius, Benson Kiniboro, Matthew Philip, Shadrach Jally, Bernadine Kasian, Naomi Sambale, Peter M. Siba, Stephan Karl, Alyssa E. Barry, Ingrid Felger, James W. Kazura, Ivo Mueller, Leanne J. Robinson

**Affiliations:** 1 Vector Borne Disease Unit, Papua New Guinea Institute of Medical Research, PO Box 378, Madang, 511 MP Papua New Guinea; 2 Division of Population Health and Immunity, Walter and Eliza Hall Institute of Medical Research, 1G Royal Parade, Parkville, VIC 3052 Australia; 3 Present Address: Department of Biomedical Sciences, Institute of Tropical Medicine, Malariology Unit, Nationalestraat 155, 2000 Antwerp, Belgium; 4 Medical Parasitology and Infection Biology, Swiss Tropical & Public Health Institute, Socinstrasse 57, 4051 Basel, Switzerland; 5 Center for Global Health and Diseases, Case Western Reserve University, 10900 Euclid Ave, Cleveland, OH 44106 USA; 6 Department of Medical Biology, University of Melbourne, Parkville, VIC 3010 Australia; 7 Present Address: School of Medicine, Deakin University, Geelong and Burnet Institute, Melbourne, VIC Australia; 8 Department of Parasites and Insect Vectors, Malaria Parasites and Hosts Unit, Pasteur Institute, 25-28 rue du Docteur-Roux, 75724 Paris Cedex 15, France; 9 Disease Elimination Program, Vector-borne Diseases and Tropical Public Health Group, Burnet Institute, 85 Commercial Rd, Melbourne, VIC 3004 Australia

**Keywords:** Malaria, *Plasmodium falciparum*, *Plasmodium vivax*, Epidemiology, Malaria control, Spatial heterogeneity, LLINs

## Abstract

**Background:**

In the past decade, national malaria control efforts in Papua New Guinea (PNG) have received renewed support, facilitating nationwide distribution of free long-lasting insecticidal nets (LLINs), as well as improvements in access to parasite-confirmed diagnosis and effective artemisinin-combination therapy in 2011–2012.

**Methods:**

To study the effects of these intensified control efforts on the epidemiology and transmission of *Plasmodium falciparum* and *Plasmodium vivax* infections and investigate risk factors at the individual and household level, two cross-sectional surveys were conducted in the East Sepik Province of PNG; one in 2005, before the scale-up of national campaigns and one in late 2012-early 2013, after 2 rounds of LLIN distribution (2008 and 2011–2012). Differences between studies were investigated using Chi square (χ^2^), Fischer’s exact tests and Student’s t-test. Multivariable logistic regression models were built to investigate factors associated with infection at the individual and household level.

**Results:**

The prevalence of *P. falciparum* and *P. vivax* in surveyed communities decreased from 55% (2005) to 9% (2013) and 36% to 6%, respectively. The mean multiplicity of infection (MOI) decreased from 1.8 to 1.6 for *P. falciparum* (p = 0.08) and from 2.2 to 1.4 for *P. vivax* (p < 0.001). Alongside these reductions, a shift towards a more uniform distribution of infections and illness across age groups was observed but there was greater heterogeneity across the study area and within the study villages. Microscopy positive infections and clinical cases in the household were associated with high rate infection households (> 50% of household members with *Plasmodium* infection).

**Conclusion:**

After the scale-up of malaria control interventions in PNG between 2008 and 2012, there was a substantial reduction in *P. falciparum* and *P. vivax* infection rates in the studies villages in East Sepik Province. Understanding the extent of local heterogeneity in malaria transmission and the driving factors is critical to identify and implement targeted control strategies to ensure the ongoing success of malaria control in PNG and inform the development of tools required to achieve elimination. In household-based interventions, diagnostics with a sensitivity similar to (expert) microscopy could be used to identify and target high rate households.

## Background

Papua New Guinea (PNG) has the highest transmission of malaria in the Western Pacific Region, where it accounted for 80% of the reported confirmed cases in 2018 [1]. The majority of the population in malaria-endemic areas have some degree of natural immunity and, therefore, the prevalence of malaria infection and incidence of morbidity are highest in young children and pregnant women. In the coastal and lowland inland areas along the north coast perennial high intensity malaria transmission is present [2–8].

East Sepik Province (ESP) has historically been an area of PNG with a high malaria burden, with a peak prevalence of 70% (by light microscopy (LM)) in children 5–9 years of age in the early 1990′s [3], whilst more recent studies indicate a lower prevalence [5, 9]. The Anopheline spp. fauna in PNG is diverse and includes 16 species [10]. The principal malaria vectors in PNG are members of the *Anopheles punctulatus* group of mosquitoes, of which *An. punctulatus*, *Anopheles koliensis*, *Anopheles farauti*, and *Anopheles farauti* 4 have been incriminated as major vectors [10–14]. The different species have different biting cycles: *An. farauti* tend to bite mostly during the early hours of the evening, and in the case of *An. punctulatus* and *An. koliensis*, peak activity takes place in the early hours of the morning [2].

*Plasmodium falciparum* and *Plasmodium vivax* are the most abundant species in PNG, though *Plasmodium malariae* and *Plasmodium ovale* are also present, usually in combination with the former two species [15]. *Plasmodium vivax* was the predominant species prior to the introduction of mass spraying and drug administration programmes in the 1950’s, which were intended to eliminate malaria [2, 16, 17], These efforts were subsequently abandoned in the 1970’s and a resurgence of *P. falciparum* followed, which remains the dominant species today [2, 17]. However, in the past decade, renewed malaria control efforts initiated by the PNG National Department of Health with support from The Global Fund to fight AIDs, TB and malaria have once again significantly reduced the burden of malaria in PNG [18–22]. This National Malaria Control Programme (NMCP) has focused on the nationwide distribution of free LLINs on a 3-yearly cycle since 2008, greater availability of prompt diagnosis and effective treatment through the introduction of rapid diagnostic tests (RDTs) and artemisinin-based combination therapy (ACT) in 2011–2012 and a behaviour change campaign, “Yumi rausim malaria”, focused on improving understanding of the best options for prevention and treatment of malaria [18, 21, 23]. ESP was among the first Provinces in PNG where the LLIN distribution campaign was implemented, which together with the availability of historical data makes it an ideal location to investigate the changing epidemiology of malaria in PNG.

While the previously reported reduction in nationwide prevalence and incidence of malaria and all-cause mortality rates in young children are significant achievements [18, 21–24], important questions related to the nature of the epidemiological transition and long-term impact of intensified control on parasite-host-vector interactions remain unresolved. Sustained reduction in malaria transmission can lead to a decrease in naturally acquired immunity and consequently, a shift in the peak burden of malaria infection and illness to older age groups and change other risk factors. A declining burden of malaria illness and high-density parasite infections can also mask a substantial community burden of low-density but gametocytaemia infections that sustain transmission [25–27]. In addition, the possible differential effects of these interventions on the various *Plasmodium* species is important to consider in co-endemic regions such as PNG, given the distinct biological differences of the two main *Plasmodium* species. While declining prevalence and morbidity have been documented on a regional level, the effects of decreasing malaria transmission on a smaller geographical scale remain to be investigated. Heterogeneities in transmission and disease burden have been described at various scales in PNG [4, 21, 25] and can be a major challenge to further reducing the burden of malaria, as hotspots can act as the source of infection for other neighbouring areas [28], and especially *P. vivax* can be resilient [25].

To investigate the impact of the renewed malaria control efforts on the epidemiology of malaria within communities, data from two cross-sectional community surveys conducted in the Maprik and Wosera-Gawi districts of East Sepik Province, PNG; one in April–May 2005, before the implementation of national LLIN distribution campaign and one in October 2012–February 2013, were directly compared. Risk factors for parasite prevalence, and spatial heterogeneity were investigated in addition to gametocytaemia and complexity/multiplicity of infection. Understanding the key factors related to heterogeneity and residual malaria transmission in PNG is critical to support the desired transition from nation-wide malaria control to sub-national elimination strategies.

## Methods

### Study design

Two cross-sectional community surveys were conducted in the Maprik and Wosera-Gawi districts in the East Sepik Province of PNG; one in April–May 2005 [9], before the implementation of national LLIN distribution campaign and one in October 2012–February 2013. The 1st round of LLIN distribution in ESP took place in 2008, followed by a second round in 2011–2012. LLINs in the study area in ESP were distributed at most 12 months prior to the 2012/13 survey: September 2011 (Brukham, Ulupu and Ilahita catchement areas) and October 2012 (Wombisa and Burui catchement areas). Introduction of a test-and-treat approach and a switch to artemether–lumefantrine (AL) as first-line treatment and formal adoption of 14 days 0.25 mg/kg primaquine for vivax-confirmed malaria occurred at the end of 2011/beginning of 2012. The Maprik and Wosera-Gawi districts, consist of an area of over 160 km^2^ with low hills, plains and riverine plains with a wet tropical climate [3, 9]. The natural vegetation is lowland hill forest that has mostly been replaced by re-growth following cultivation and wide grasslands on the plains near the Sepik River. The people in this area live in villages of hundred to several hundred individuals and the villages are sometimes divided in geographically distinct hamlets. The majority of the people live from subsistence farming. There are several government health centres, church health centres and smaller aid posts in the area and the referral hospital is in Maprik.

All eligible and willing residents of households in the local communities in the study area were invited to participate and following written informed consent, demographic information (age, gender, familial relationship, bed net use), history of febrile illness, household or village location by GPS receiver and blood samples were collected. The design of both studies was similar, and details of the 2005 study have been previously described [9]. All details of the recent cross-sectional are described below. In the 2005 study questions related to bed nets were not directed to insecticide treated nets (ITNs) or LLINs in particular, and are likely to be untreated nets. In 2012–2013, after the start of the large-scale LLIN campaigns, questions were asked specifically about LLINs and not ITNs or untreated nets. Therefore, when variables are reported where the 2005 study is included, the term bed nets will be used, while in the 2012–2013 study the term LLINs can be applied.

Capillary blood (250–300 μL) was collected into K + EDTA microtainers, thick/thin films were prepared and haemoglobin levels were measured (Hemocue). The collected blood was centrifuged, the plasma removed and stored at − 80 °C, and the red cell pellet stored at -20 °C until DNA extraction. If febrile illness was reported, a rapid malaria diagnostic test (Carestart Pf/Pan) was performed and those positive by RDT treated with artemether-lumefantrine (Coartem). For RNA preservation, 50 μL of whole blood was immediately transferred to a tube containing 250μL of RNAProtect (Qiagen) and stored at -80 °C within 8 h of collection until RNA extraction.

### *Plasmodium* spp. detection

Giemsa-stained thick and thin films from the 2005 survey were examined by LM (minimum of 200-high powered fields) by two independent experienced microscopists, with discrepancies adjudicated by a third independent microscopist, as previously described [9]. All slides collected during the 2012/13 study, were similarly examined once by experienced microscopists. Slides from LM and/or PCR positive individuals and a random selection of negative slides were re-examined by an independent microscopist with discrepancies adjudicated by a third WHO-certified Level 1 or 2 microscopist. Parasite densities were calculated from the number of parasites per 200 or 500 white blood cells (WBCs) (depending on parasitaemia) and an assumed total peripheral WBC count of 8000/μL [29], with the final density taken as the geometric mean of the two values.

The presence of parasite DNA in all blood samples was investigated by molecular methods (MM, i.e. qPCR or LDR-FMA). In 2005 DNA was extracted from red blood cell pellets using QIAmp 96 DNA Blood kits (Qiagen) and a post-PCR, ligase detection reaction/microsphere assay (LDR-FMA) was used to determine the presence of *P. falciparum, P. vivax, P. malariae* and *P. ovale* [9, 30]. In the 2012/13 survey, DNA was extracted from the equivalent of 200 μL whole blood using the Favorgen 96-well Genomic DNA Extraction Kit (Favorgen) following the manufacturer’s instructions and eluted in 200 μL. Initially a generic quantitative PCR (QMAL) that amplifies a conserved region of the 18S rRNA gene was run on all samples [31]. Species-specific quantitative PCRs (qPCR) were then performed on all positive samples as described [32]. The qPCR was previously directly compared to the LDR-FMA and assays are approximately equivalent in sensitivity and specificity [32]. Copy numbers were quantified based on serial dilutions of plasmid controls run in parallel.

### Gametocyte detection

The number of observed *P. falciparum* (2005 and 2012/13) and/or *P. vivax* (2012/13 only) gametocytes were recorded separately from asexual stages during microscopic examination of all blood slides. Gametocyte detection by qRT-PCR was performed on samples from 2012/13: RNA was extracted from all *P. falciparum* and/or *P. vivax* qPCR-positive samples using the Qiagen RNeasy plus 96 kit, according to the manufacturer’s procedures. A genomic DNA removal by gDNA eliminator columns and DNase (Qiagen) step was included in the procedure. Absence of gDNA was confirmed by qPCR and presence of parasite RNA after extraction was verified by qRT-PCR with the same primers and probe as the QMAL qPCR described above. *P. falciparum* and *P. vivax* gametocytes were detected by qRT-PCR of the highly expressed gametocyte markers *pfs25* and *pvs25* as previously described [31]. The limit of detection of the *pfs25* and *pvs25* qRT-PCRs in the laboratory set up in PNG, were 6 copies/transcript per reaction for both assays.

### Genotyping

To genotype *P. falciparum* and *P. vivax* LDR-FMA/qPCR positive samples, high-resolution *Pfmsp2*, *Pvmsp1f3* and PvMS2 genotyping was performed as previously described [33, 34]. Briefly, for *P. falciparum* a nested multiplex PCR approach was used to amplify 3D7 and/or FC27 family alleles of *Pfmsp2* using family-specific primers labelled with a fluorescent dye, or a multiplex primary PCR was used to amplify the markers *Pvmsp1F3* and PvMS2 with fluorescently labelled primers. The PCR products were analysed by 1.5% agarose gel electrophoresis and the number and size of alleles were then determined by capillary electrophoresis using a 23 ABI 3730XLs platform (Macrogen, Korea) with the internal size standard GSLIZ500 and data were subsequently analysed using GeneMarker 2.4.0 demo version (SoftGenetics). The multiplicity of infection (MOI) for *P. falciparum* was defined as the number of *Pfmsp2* alleles counted within a sample, and *P. vivax* MOI was defined by the number of *Pvmsp1f3* or PvMS2 alleles, whichever was higher.

### Data analysis

Raw data from the 2005 survey [9] was reanalysed to compare prevalence and predictors with data from the 2012/13 survey. Clinical malaria cases were defined as febrile illness (current or previous 48 h) in the presence of *P. vivax* or *P. falciparum* asexual parasites by LM (any density). Symptomatic infections were defined as febrile illness (current or previous 48 h) in the presence of parasites as detected by LM and/or MM. Parasite densities were log transformed and the geometric mean per μL whole blood are reported. Four categories were used to describe anaemia: non-anaemia, mild, moderate or severe, which were defined based on haemoglobin concentrations (measured by Hemocue) and stratified by age and gender as per the WHO recommendations (Additional file 1) [35]. On average, there were 3.7 people in a household (3.8 in 2005; 3.5 in 2012–2013). A high-infection rate household was defined on the basis of having more than 50% of household members with a *Plasmodium* infection (determined by molecular method).

Differences between the two studies were investigated using Chi square (χ^2^) and Fischer’s exact tests for categorical characteristics and Student’s t-test for normally-distributed continuous variables. Tests were two-tailed and the confidence level was set at 95%. Univariable analysis of factors associated with *P. falciparum* or *P. vivax* infection (determined by MM and/or LM) and high rate household were conducted using logistic regression. Multivariable logistic regression models were built with variables with p < 0.15 in univariate analysis and variables were selected using likelihood methods for models with minimal Akaike information criterion (AIC). Analyses were performed using Stata 12. Genetic diversity analyses were done using FSTAT software version 2.9.3.2 to define allele frequencies, and the expected heterozygosity (*He*). Household, village and health facility location data were collected using a handheld GPS receiver (Garmin GPSmap62sc). Maps were constructed with collected GPS coordinates using ARC GIS Pro 10.4

## Results

### Study characteristics

A total of 2744 participants from 15 villages in five distinct geographical areas of East Sepik Province participated in the 2005 cross-sectional survey. Of these 121 (4.4%) were excluded because of missing demographic or LM data, while insufficient finger-prick blood sample for LDR-FMA analysis led to exclusion of an additional 96 (3.5%) individuals. Overall 2527 participants from 659 households with complete demographic and infection status data were available for comparison with 2012/13 data. A total of 2500 participants in 14 villages from the same areas participated in the 2012/13 cross-sectional survey. Of these, 14 (0.6%) were excluded from the analysis because of missing LM results. Overall, 2486 participants from 704 households with complete demographic and infection status data were available.

The two studies were very comparable in their design, however, they differed in the demographic and clinical characteristics of the participants (Table 1 and Additional file 2). Median age in 2005 was 17 years (range 2.5 weeks–80 years) and 22 in 2012/13 (range 6 months – 68 years). There were three villages from the 2005 study that weren’t surveyed in 2012/2013, instead two other villages from the same areas were included. As expected after two rounds of LLIN distribution (2008 and 2011/12), significantly more participants reported using LLINs in 2012/2013 (94.9%) compared to bed nets in 2005 (88.3%) (Table 1). The prevalence of clinical episodes of malaria (*P. falciparum* and/or *P. vivax*) had dropped from 3.6% to 2.3% (p = 0.007). There was a lower prevalence of anaemia in 2012/13, and accordingly, mean haemoglobin levels were significantly higher in 2012/13 (11.1 ± 1.8 g/dL) compared to 2005 (10.5 ± 1.7 g/dL; p < 0.001) (Table 1). In addition, a higher proportion of individuals reported a current or recent febrile illness in 2012/13 (14.4% vs 7.4% p < 0.001).

**Table 1:** Summary of demographic and clinical characteristics of study participants

	2005		2013		*p*-value
	n	%	n	%	
Number of participants	2527		2486		
Gender (female)	1331	52.7	1438	57.8	*< 0.001*
Age in years (median [range])	17 [0–80]		22 [0–68]		*< 0.0001*
Reported use of bed nets/LLINs	2231	88.3	2348	94.9	*< 0.001*
History of recent malaria infection	409	16.3	137	5.5	*< 0.001*
History of recent malaria treatment	195	8.0	145	5.87	*0.003*
Anaemia	2131/2486	85.7	1719/2382	72.2	*< 0.001*
Current or recent reported febrile illness	186	7.4	356	14.4	*< 0.001*
Clinical malaria (due to *P. falciparum* and/or *P. vivax*)	92	3.6	58	2.3	*0.007*

A more detailed comparison can be found in Additional file 2. Italics *p*-values are considered statistically significant (*p *< 0.05)

### Prevalence of *Plasmodium* spp. infections in 2005 compared to 2012/13

The prevalence of malaria infections decreased from 44.4% to 8.3% by LM and 73.0% to 12.2% by molecular methods (MM) (Table 2). The species composition remained fairly similar, with *P. falciparum* the predominant species in both years (75.2% of all infections in 2005 and 70.0% in 2012/13 by MM, p = 0.052, χ^2^), followed by *P. vivax* (48.9% of all infections in 2005, and 45.9% in 2012/13 by MM, p = 0.335, χ^2^). The prevalence of both species dropped with similar proportions; in other words, for both species the prevalence in 2012/2013 was roughly 16% of the prevalence in 2005 (Table 2). A lower proportion of infections were *P. malariae* (p < 0.001, χ^2^) in 2012/13 compared to 2005 while the proportion of *P. ovale* by MM remained similar. The majority of mixed infections in both years consisted of *P. falciparum* - *P. vivax* co-infections, followed by *P. falciparum* with *P. malariae* and/or *P. ovale*, as determined with MM. The proportion of *P. falciparum* infections that were sub-microscopic decreased significantly from 2005 (47.2%) to 2012/13 (37.4%; p = 0.006), but not for *P. vivax* (60.4% vs 54.0%; p = 0.139, χ^2^). The geometric mean density of parasites by microscopy was similar in both years. There was a significant increase in the proportion of malaria infections with any species that were symptomatic, increasing from 7.0% in 2005 to 25.3% in 2012/13 (p < 0.001, χ^2^), as there was a higher rate of people reporting febrile illness in 2012–2013 (Table 1).

**Table 2:** Prevalence of *Plasmodium* spp. infections of study participants

	2005 (n = 2527)		2013 (n = 2486)		*p*-value
Parasite detection by light microscopy					
All species	1121/2527	44.4%	206/2486	8.3%	*< 0.001*
*P. falciparum*	756/2527	29.9%	139/2486	5.6%	*< 0.001*
Asexual parasites	730/2527	28.9%	128/2486	5.2%	*< 0.001*
Gametocytes	80/2527	3.2%	33/2486	1.3%	*< 0.001*
Geometric mean density (SD)	464.2	(6.8)	487.3	(9.3)	*0.798*
*P. vivax*	368/2527	14.6%	69/2486	2.8%	*< 0.001*
Asexual parasites			69/2486	2.8%	
Gametocytes			19/2486	0.8%	
Geometric mean density (SD)	193.2	(4.2)	218.5	(5.6)	*0.531*
*P. malariae*	99/2527	9.0%	6/2486	0.2%	*< 0.001*
*P. ovale*	0/2527	0.0%	2/2486	0.1%	*0.246*
Mixed any species	100/2527	6.5%	9/2486	0.4%	*< 0.001*
Parasite detection by molecular method					
All species	1844/2527	73.0%	303/2486	12.2%	*< 0.001*
*P. falciparum*	1387/2527	54.9%	212/2486	8.5%	*< 0.001*
Gametocytes	NA		94/2486	3.8%	
*P. vivax*	901/2527	35.7%	139/2486	5.6%	*< 0.001*
Gametocytes	NA		23/2486	0.9%	
*P. malariae*	338/2527	13.4%	28/2486	1.1%	*< 0.001*
*P. ovale*	121/2527	4.8%	29/2486	1.2%	*< 0.001*
Mixed any species	723/2527	28.6%	93/2486	3.7%	*< 0.001*
Mixed with *P. falciparum*	683/2527	27.0%	86/2486	3.5%	
Mixed non-*P. falciparum*	40/2527	1.6%	7/2486	0.3%	
Symptomatic infections of any species	132/2527	5.2%	80/2486	3.2%	*< 0.001*

Italics *p*-values are considered statistically significant (*p *< 0.05)

The proportion of *P. falciparum* infected individuals with gametocytes detected by LM increased from 10.6% in 2005 to 23.7% in 2012/13 (Table 2). The presence of *P. falciparum* and *P. vivax* gametocytes in the 2012/13 survey was also investigated with a qRT-PCR detecting gametocyte specific RNA, and the proportion of *P. falciparum* infections with gametocytes (51.4%) by that method was significantly higher than with LM (p < 0.001, χ^2^). *Plasmodium vivax* gametocytes detected by LM were not recorded in 2005. In 2012/13, the prevalence of *P. vivax* gametocytes was 0.8% by LM and 0.9% by qRT-PCR (Table 2).

### Comparing risk factors for *Plasmodium* spp. infections and illness

Village of residence was a risk factor for parasite infection in both surveys (Table 3). In addition, moderate or severe anaemia was a strong predictor for *P. falciparum* infection in both surveys, but not for *P. vivax* (Table 3). While males were more likely to be *P. falciparum* infected than women in 2005 (aOR 1.2 [1.01–1.4], p = 0.04), there was no difference in 2012–2013. In 2012–2013, current or recent fever was a predictor of *P. falciparum* infection (aOR 2.0 [1.3–3.1], p = 0.005). In contrast, participants with recent/current febrile illness were more likely to be *P. vivax* infected in 2005, but not in 2012/13 (Table 3), and recent anti-malarial treatment was associated with reduced risk of *P. vivax* in 2005. Infection with the other species was a predictor for both *P. falciparum* and *P. vivax* in 2012–2013 (Table 3).

**Table 3:** Multivariable predictors of *P. falciparum* (A) and *P. vivax* (B) infections

A. Predictors of *P. falciparum* infections								
	2005 (N = 2486)				2012–2013 (N = 2370)			
	% Pf	aOR	95% CI	*p*-value	% Pf	aOR	95% CI	*p*-value
Female	55.5%	1.0		*0.040*				
Male	57.3%	1.2	[1.01–1.4]					
Anaemia				*< 0.001*				*0.0029*
No	49.3%	1.0			3.8%	1.0		
Mild	51.2%	1.2	[0.95–1.6]		5.0%	1.1	[0.6–2.0]	
Moderate	60.0%	1.8	[1.4–2.3]		12.0%	1.9	[1.1–3.2]	
Severe	71.7%	3.4	[2.0–5.6]		28.1%	3.9	[1.6–9.8]	
Age group				*< 0.001*				0.103
0–3	25.8%	1.0			6.0%	1.0		
> 3–6	44.4%	2.9	[1.8–4.4]		8.7%	3.2	[0.7–15.3]	
> 6–9	59.5%	5.1	[3.3–7.6]		12.0%	4.6	[1.0–22.0]	
> 9–12	70.2%	8.2	[5.2–12.]		10.7%	4.1	[0.9–19.6]	
> 12–20	73.6%	12.9	[8.3–19.]		9.2%	7.1	[1.5–34.5]	
> 20	54.1%	4.7	[3.1–6.8]		6.3%	4.0	[0.9–18.1]	
Current or recent reported febrile illness								*0.0050*
No					6.8%	1.0		
Yes					14.8%	2.0	[1.3–3.1]	
*P. vivax* infected by qPCR								*< 0.001*
No					5.6%	1.0		
Yes					48.1%	5.6	[3.4–9.1]	
Village				*< 0.001*				*< 0.001*
5. Ilahita 3	57.4%	1.0			2.1%	1.0		
6. Ilahita 4	55.0%	1.0	[0.60–1.5]		3.0%	1.4	[0.3-6.0]	
7. Sunuhu	81.9%	3.8	[2.2–6.2]		51.4%	26.2	[7.8–88.1]	
4. Urita	61.0%	1.1	[0.71–1.8]		5.0%	1.7	[0.4–6.9]	
3. Salata	NA				6.1%	2.7	[0.7–9.9]	
2. Waragom	68.6%	1.6	[0.97–2.5]		16.7%	8.0	[2.2–29.4]	
12. Jama	38.8%	0.4	[0.2–0.6]		3.9%	1.8	[0.4–7.8]	
13. Sengo	54.3%	0.8	[0.48–1.2]		7.5%	4.2	[1.2–14.5]	
14. Maiwi	48.0%	0.6	[0.35–0.9]		13.4%	6.6	[1.7–25.8]	
15. Malba 1	49.7%	0.7	[0.47–1.1]		1.3%	0.6	[0.1–2.9]	
16. Malba 2	58.6%	1.2	[0.73–1.8]		3.6%	2.2	[0.6–8.6]	
17. Yenigo	50.3%	0.9	[0.55–1.3]		0.6%	0.4	[0.0–3.7]	
10. Wapindumaka	NA				3.8%	2.1	[0.5–8.5]	
11. Wombisa	53.5%	0.8	[0.51–1.3]		1.0%	0.5	[0.08–3.0]	
8. Wapin	49.3%	0.8	[0.47–1.2]		NA			
1. Bonohi	56.4%	1.1	[0.68–1.7]		NA			
9. Bangeleko	59.5%	1.0	[0.63–1.6]		NA			

B. Predictors of *P. vivax* infections								
	2005 (N = 2441)				2013 (N = 2324)			
	%	aOR	95% CI	*p*	%	aOR	95% CI	*p*
Recent reported anti-malarial treatment				*< 0.001*				
No	38.3%	1						
Yes	22.1%	0.4	[0.3–0.6]					
Current or recent reported febrile illness				*0.006*				
No	37.9%	1.0						
Yes	25.6%	0.6	[0.4–0.9]					
Female	34.8%	1.0		0.077				
Male	39.5%	1.2	[0.98–1.4]					
Age group				*< 0.001*				*0.0030*
0–3	24.3%	1.0			6.5%	1.0		
> 3–6	49.0%	3.0	[1.9–4.7]		7.5%	1.4	[0.3–5.8]	
> 6–9	48.7%	2.8	[1.9–4.3]		10.7%	2.2	[0.5–9.1]	
> 9–12	53.1%	3.4	[2.2–5.3]		11.7%	2.8	[0.7–11.8]	
> 12–20	37.1%	1.7	[1.1–2.6]		5.8%	1.3	[0.3–5.2]	
> 20	29.7%	1.1	[0.8–1.7]		3.8%	0.9	[0.2–3.7]	
*P. falciparum* infected by qPCR								*< 0.001*
No					3.3%	1.0		
Yes					32.7%	4.9	[3.1–7.8]	
Village				*0.0012*				*< 0.001*
5. Ilahita 3	38.6%	1.0			2.1%	1.0		
6. Ilahita 4	37.8%	1.1	[0.7–1.7]		2.4%	1.1	[0.2–5.1]	
7. Sunuhu	40.3%	1.1	[0.7–1.8]		36.0%	11.9	[3.5–40.4]	
4. Urita	46.9%	1.3	[0.8–2.1]		9.0%	4.2	[1.2–14.9]	
3. Salata	NA				5.3%	2.4	[0.7–8.6]	
2. Waragom	42.0%	1.1	[0.7–1.7]		8.4%	3.5	[0.9–13.7]	
12. Jama	29.1%	0.6	[0.4–1.0]		3.1%	1.3	[0.3–6.1]	
13. Sengo	26.2%	0.5	[0.3–0.8]		4.4%	1.8	[0.5–6.3]	
14. Maiwi	36.4%	0.8	[0.5–1.3]		2.9%	0.9	[0.1–5.4]	
15. Malba 1	44.9%	1.2	[0.7–1.9]		1.6%	0.7	[0.2–3.4]	
16. Malba 2	42.2%	1.1	[0.7–1.8]		1.7%	0.7	[0.1–3.4]	
17. Yenigo	36.9%	0.9	[0.5–1.4]		NA			
10. Wapindumaka	NA				0.5%	0.2	[0.02–2.0]	
11. Wombisa	34.9%	0.8	[0.5–1.3]		0.5%	0.2	[0.02–2.3]	
8. Wapin	29.9%	0.6	[0.4–1.1]		NA			
1. Bonohi	34.7%	0.8	[0.5–1.3]		NA			
9. Bangeleko	33.1%	0.7	[0.4–1.1]		NA			

% indicate proportion infected with *P. falciparum* or *P. vivax* detected by LDR-FMA (2005) or qPCR (2013) and/or LM within the designated group

*aOR* multivariable adjusted odds ratio, *CI* confidence interval

Italics *p*-values are considered statistically significant (*p *< 0.05)

**Fig. 1:**
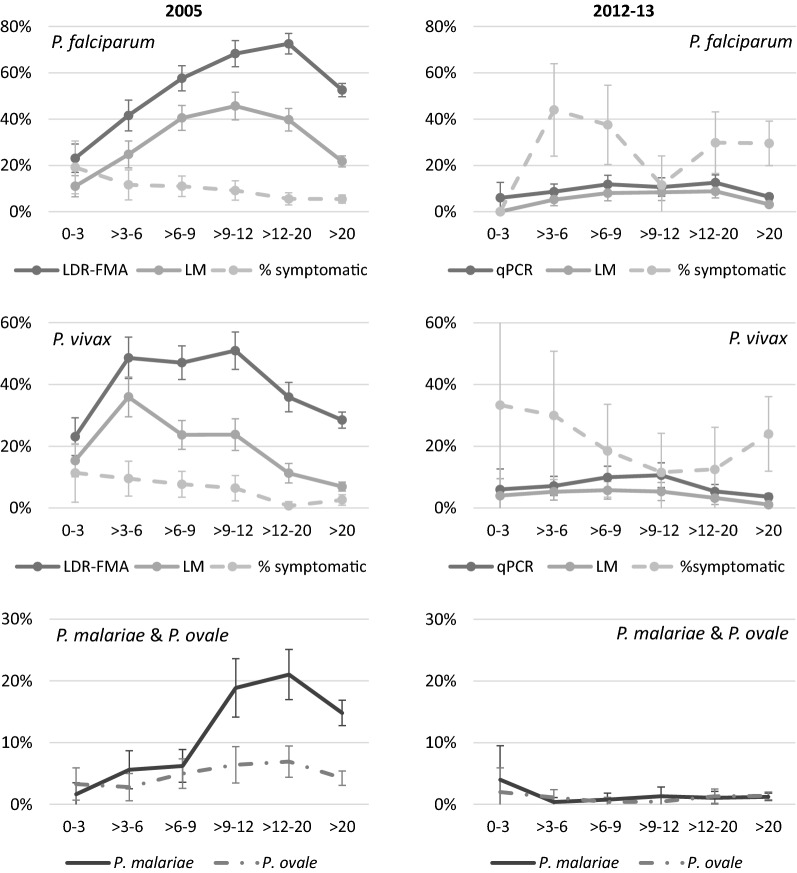
Age distributions of malaria prevalence. Top 4 panels: *P. falciparum* or *P. vivax* prevalence by LDR-FMA or qPCR, prevalence by light microscopy and proportion symptomatic *P. falciparum* or *P. vivax* infections. Bottom panels: prevalence of *P. malariae* and *P. ovale*. Error bars represent 95% confidence intervals. Left 2005 survey, right 2012/13 survey

Although there was a considerable reduction in the overall prevalence of malaria between 2005 and 2012/13, there was no obvious shift in the age of peak prevalence of *P. falciparum* and *P. vivax* infections (Fig. 1, Table 3). However, a more uniform distribution of infections across all age groups was observed in 2012/13 compared to 2005, with age no longer a significant predictor of *P. falciparum* infection in 2012/13 (Table 3). *Plasmodium falciparum* infections were most frequent in older children, especially in 2005 (12–20 years by MM in 2005 and 6–20 years by MM in 2012–2013) (Fig. 1). *Plasmodium vivax* prevalence was highest in younger children (3–12 years by MM) compared to *P. falciparum* (Fig. 1 and Table 3). Compared to 2005, an increase in the proportion of symptomatic infections was seen in most age groups in 2012–2013 (Fig. 1), and age was not a significant predictor of symptomatic infections in 2012–2013 (logistic regression, p > 0.05). The increase in proportion of symptomatic infections in 2012/13 is especially large in children and adults above 12 years; fivefold in the case of *P. falciparum* and 9 to 18-fold in the case of *P. vivax* infections. None of the *P. falciparum* infections observed in 2012/13 between the ages of 0–3 years were accompanied with fever (Fig. 1).

**Fig. 2:**
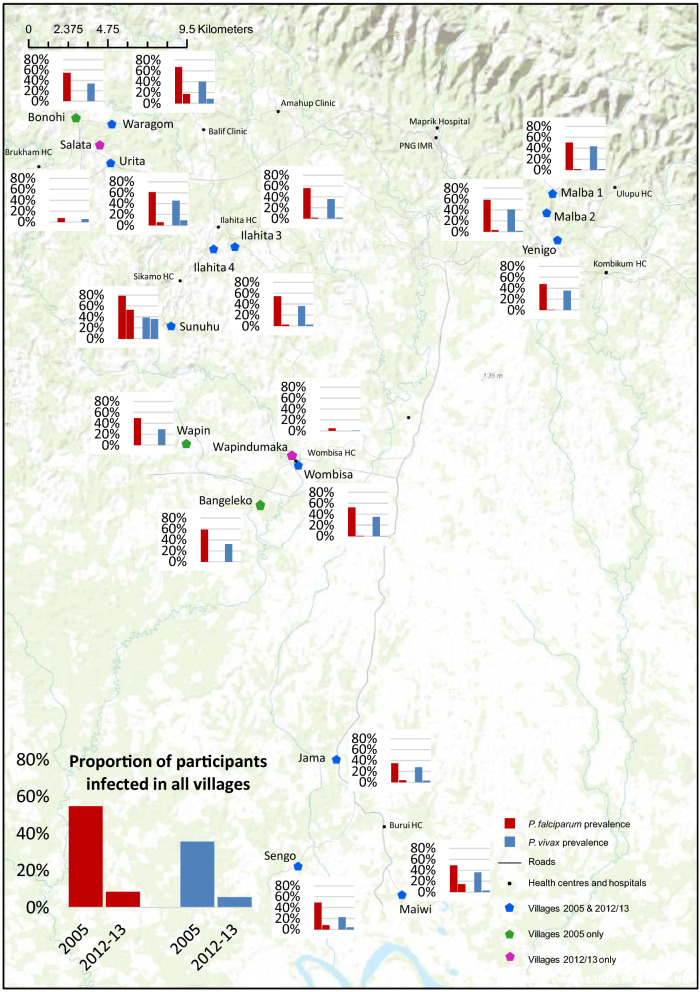
Map of study area with village-specific malaria prevalence in 2005 and 2013. Red bars indicate *P. falciparum* prevalence and blue bars *P. vivax* prevalence. Villages with a blue symbol (pentagon) were included in both surveys, villages with a green symbol were included in the 2005 survey only and villages with a purple symbol were included in the 2012–2013 survey only

The decrease in the prevalence of *P. falciparum* and *P. vivax* was much greater in some villages than in others, indicative of heterogeneous transmission (Fig. 2). Of the 12 villages sampled in both years, there were 6 villages where the malaria prevalence by MM in 2012/13 had declined by more than 90% compared to 2005, 5 where the prevalence had reduced by 73–88% and one (Sunuhu) where the prevalence in 2012/13 had decreased by only 28%. These effects were quite similar for both *P. falciparum* and *P. vivax,* while in 1 village in 2012/13 no *P. vivax* infections were detected at all.

### Parasite genetic complexity in 2005 compared to 2012/13

The majority of *P. falciparum* infections detected by LDR-FMA in 2005 and qPCR in 2012/13 were successfully genotyped (63.0% and 77.8%, respectively). Similarly, *P. vivax* infections were successfully genotyped by at least one of two markers *Pvmsp1f3* and PvMS2 (63.5% in 2005 and 64.7% 2012/13). The total number of different alleles detected by the markers was lower in 2012/13 than in 2005, but the most common alleles were similar between the 2 years (Additional file 3). The mean multiplicity of infection (MOI) decreased from 1.8 to 1.6 for *P. falciparum* (p = 0.08, Mann–Whitney U test) and from 2.2 to 1.4 for *P. vivax* (p < 0.001, Mann–Whitney U test). However, the proportion of *P. falciparum* multiple clone infections (MOI > 1) remained constant (45.4% in 2005 vs 42.4% in 2012/13; p = 0.478, χ^2^), while the proportion of *P. vivax* multiple clone infections decreased from 58.2% in 2005 to 31.1% in 2012/13 (p < 0.001, χ^2^). In addition, 54% of *P. vivax* single clone infections were sub-microscopic in 2012/13, and MOI was significantly associated with sub-microscopic *P. vivax* infections in both years (p < 0.001, logistic regression). Despite the significant decrease in prevalence, the overall genetic diversity (*He*) was high in both years for both species (Table 4). Despite large differences in prevalence in 2012–2013, MOI and *He* were very similar across catchment areas (Table 4) and villages, and diversity seemed lowest in the highest prevalence area.

**Table 4:** Genetic diversity, prevalence and multiplicity of infection of *P. falciparum* and *P. vivax* infections at the health centre catchment area level

	2005			2012/13		
	Prevalence (%)	MOI	*He*	Prevalence	MOI	*He*
*Pfmsp2*						
Overall	54.9	1.8	0.94	8.5	1.6	0.91
Ilahita	63.8	2.2	0.93	21.6	1.6	0.88
Brukham	60.8	1.9	0.94	8.8	1.4	0.93
Burui	43.4	1.4	0.95	7.9	1.5	0.94
Ulupu	52.2	1.7	0.92	1.9	1.5	0.97
Wombisa	53.8	1.7	0.94	2.5	1.6	0.83
*Pvmsp1f3*						
Overall	35.7	2.1	0.89	5.6	1.4	0.90
Ilahita	37.2	2.7	0.91	15.0	1.4	0.85
Brukham	39.8	2.3	0.92	7.2	1.3	0.92
Burui	28.4	1.6	0.88	3.8	1.3	0.94
Ulupu	39.9	2.1	0.88	1.2	1	
Wombisa	32.4	1.9	0.88	0.5	N/A	
PvMS2						
Overall	35.7	1.8	0.91	5.6	1.3	0.90
Ilahita	37.2	2.3	0.91	15.0	1.2	0.91
Brukham	39.8	1.8	0.91	7.2	1.2	0.92
Burui	28.4	1.7	0.93	3.8	1.4	0.87
Ulupu	39.9	1.8	0.91	1.2	1.8	
Wombisa	32.4	1.6	0.91	0.5	N/A	

*MOI* mean multiplicity of infection, *He* expected heterozygosity. Diversity calculation for *P. vivax* in Ulupu in 2012–2013 was not calculated due to small sample size

**Fig. 3:**
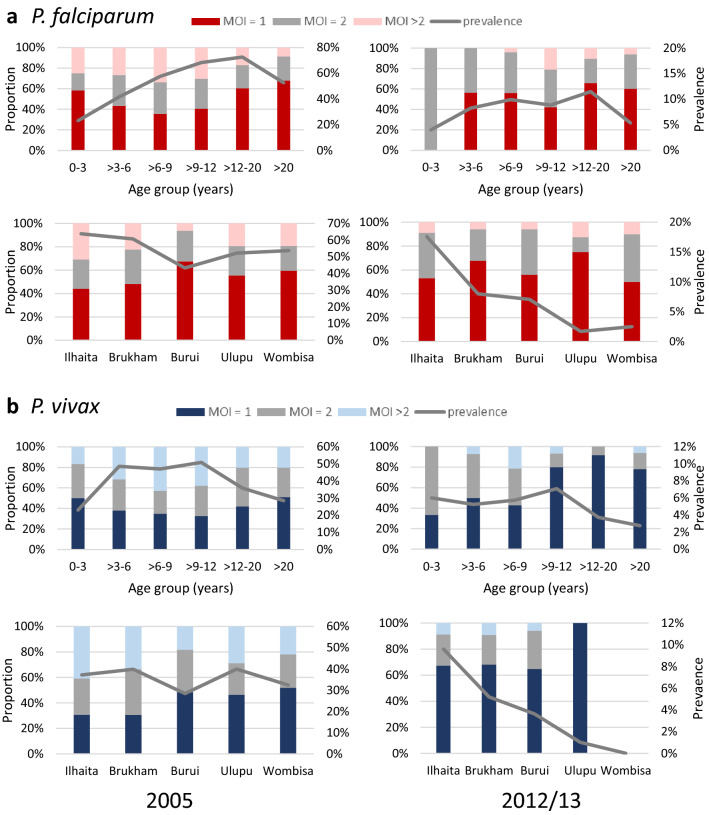
Proportion of single and multiple clone *P. falciparum* and *P. vivax* infections. **a***P. falciparum* infection by age group and catchment area; **b***P. vivax* infection by age group and catchment area; Left 2005, right 2012/13. Red/grey: *P. falciparum*, Blue/grey: *P. vivax*. *MOI* multiplicity of infection

In both years, the majority of *P. falciparum* infections in individuals over 12 years were single clone infections, whereas between the ages of 3 to 12 years more multiple clone infections were found (Fig. 3a). Single clone *P. vivax* infections were similarly distributed between all age groups in 2005, however, in 2012/13, there was a significantly higher proportion of *P. vivax* single clone infections in ages above 9 years (Fig. 3b). The proportion of multiple clone *P. falciparum* and *P. vivax* infections are quite evenly distributed in the different areas, despite differences in prevalence between the villages in both years (Figs. 3c, d).

### Heterogeneity of infection at the household level

Overall, considerably fewer households were affected by malaria infections in 2012/13 than in 2005. In 2005, 96.5% of all households had 1 or more individuals with an infection (detected by LM and/or MM), while in 2012–2013 only 27.8% of households had an individual with a LM and/or MM-detectable infection (χ^2^ p < 0.001). As expected, the proportion of households that were infected in 2012–2013 was highest in villages with relatively higher prevalence of infection. In 2005, the majority of households had 2 or more individuals infected (81.1% of all infected households), whereas in 2012–2013, the majority of infected households had only 1 individual infected (61.7% of infected households) (Fig. 1). Although self-reported LLIN/bed net ownership or use was not a significant predictor of malaria infection or illness at the individual level, household-stratified analysis revealed that a higher prevalence of malaria in a village (any species by LM and/or PCR) was significantly associated with lower proportion of self-reported bed net/LLIN use in the household in both years (p ≤ 0.004, linear regression corrected for mean age).

In 2005, 76.4% of the infected households were high-infection rate household (more than 50% of individuals in the household infected), whereas in 2012–2013, this had decreased to 27.6% (p > 0.001) and the proportion of high-infection rate households (of all infected households) was associated with prevalence in the village in both years (linear regression, p < 0.001). A higher proportion of reported bed net use in the household was associated with a reduced risk of the household being a high rate household in 2005 (Table 4), but not in 2012–2013 when reported LLIN use was very high (95%, see Additional file 2). Predictors of high-infection rate households in 2012/13 were households with the presence of clinical cases, microscopic infections and/or gametocyte carriers (Table 5).

**Table 5:** Multivariable predictors of households with high proportion of household members with MM-detectable infections (> 50%), adjusted for mean age in the household

Predictors of households with a high proportion of MM-detectable infections			
2005 (Nhh = 659)	aOR	[95% CI]	*p*-value
Proportion of hh members reporting use of bed net	0.4	[0.2–0.8]	*0.012*
Moderate or severe anaemia in the hh	1.0	[0.6–1.7]	0.95
Microscopic infections in the hh	5.9	[3.8–9.1]	*< 0.001*
Gametocyte carrier (by LM) in the hh	3.7	[1.4–9.4]	*0.007*

2012–2013 (Nhh = 430)	aOR	[95% CI]	*p*-value
Clinical case in the hh	2.6	[1.0–6.9]	0.05
Microscopic infections in the hh	4.8	[1.4–16.4]	*0.012*
Gametocyte carrier (by LM) in the hh	3.8	[1.4–10.6]	*0.011*
Ilhaita_4 (reference)	1.0		
Sunuhu	34.9	[4.1–299.3]	*0.001*
Urita	1.7	[0.1–30.1]	0.733
Salata	7.2	[0.8–65.5]	0.078
Waragom	4.3	[0.4–51.2]	0.246
Jama	1.7	[0.1–30.2]	0.725
Sengo	1.8	[0.2–19.5]	0.610
Maiwi	7.7	[0.6–107.8]	0.129
Ilhaita_3	No high rate households		
Malba_1	No high rate households		
Malba_2	No high rate households		
Yenigo	No high rate households		
Wapindumaka	No high rate households		
Wombisa	No high rate households		

*CI* confidence interval, *hh* household, *LM* light microscopy, *MM* molecular method, *Nhh* number of households

Italics *p*-values are considered statistically significant (*p *< 0.05)

## Discussion

In the historically high transmission region of ESP in PNG, a dramatic change in the burden and epidemiology of malaria was observed over the period of time coinciding with the scale-up of control via nationwide LLIN distributions and strengthening of malaria diagnosis and treatment at peripheral health facilities. Overall *Plasmodium* prevalence as detected by LM and/or MMs has declined from 74.2% in 2005 to 12.8% in 2012/13, a substantially sharper decline than has been observed in another study area along the North Coast of Papua New Guinea [36]. Alongside the reduction in prevalence and complexity of infections, a shift towards a more uniform distribution of infections and illness across age groups but greater heterogeneity in transmission across the study area and within the study villages was observed.

Heterogeneity in malaria prevalence between villages is not a new phenomenon [3, 4, 37–39]. In the 2012–2013 study, transmission in some villages had not declined as much as in others, increasing this difference between and within villages. In those areas with higher prevalence, reported use of LLINs was lower and anaemia and fever were more prevalent. While coverage of bed nets was already high in this area in 2005 (88.3%), the majority of nets at that time were untreated nets, whereas in 2012–2013 most people should have had access to LLINs, and questions specified LLINs. In the village with the highest prevalence, Sunuhu, LLIN use was lowest, which could be a major factor contributing to the high level of transmission in that village. The use of insecticide-treated nets (ITNs) has been shown in many studies to be effective in reducing mortality and morbidity from malaria [40–42]. In addition, it is thought that use of ITNs leads to community-level effects, where the majority of the population (even those not using ITNs) are protected when ITN coverage in the community is high, due to reduction in the number of infected mosquitoes and mosquito survival [43–46]. This effect has, however, not been well quantified and impact of LLINs varies with the coverage rate. In addition, the required coverage might be different for different areas, depending on local factors such as the anopheline density, species composition and both vector and human behaviour [47].

Renewed political and financial will to strengthen malaria control at the beginning of the millennium, resulted in the PNG National Department of Health launching a new campaign to quickly achieve high levels of LLIN ownership and usage. Nationwide free LLIN distribution took place between 2004 and 2009 and resulted in a significant increase in ownership of bed nets (any type 80.1%; LLINs 64.6%) [20]. Despite this increase, reported LLIN use remained low (32.5%), and the majority of people not using nets reported not having access to (unoccupied) nets [20]. A second round of country-wide LLIN distribution was conducted between 2010 and 2014 to cover the gaps in mosquito net coverage [21]. LLINs in the study area in ESP were distributed 12 months prior to this 2012/13 survey (September 2011 and October 2012) and a new round of LLIN distribution occurred in 2014/2015 (personal communication, NMCP/Rotarians Against Malaria). Large-scale LLIN distribution campaigns were performed in ESP earlier than in many other provinces with a high malaria burden, and LLIN coverage in ESP seems to be higher than on the North Coast area in general [21, 36], likely due to a higher density of nuisance biters encouraging greater use of LLINs. The substantial impact of LLINs on transmission in this area might also be due to the fact that transmission in ESP is predominantly driven by *An. punctulatus*, which feeds later at night (after midnight/early morning) and equally indoors and outdoors, making it highly susceptible to LLINs. A recent study reported an increase in prevalence of *An. punctulatus* and a decrease of *An. koliensis*, another late biter, in an adjacent area in East Sepik province, as well as a shift to earlier mean biting times of *An. punctulatus* in addition to significantly reduced man-biting-rates and annual entomological inoculation rates after the LLIN campaign [48]. Proximity to vector-breeding sites is related to the risk of malaria and can also be a main driver of heterogeneity [49–51], but was not investigated in the current study.

Cultural factors, socio-economic status and education level play an additional role in risk of infection and can vary across villages and households, as well as human behavioural and genetic factors [9, 21, 28, 52–56]. The time and manner of LLIN distribution in these areas can play a role in their availability, use and quality/age of the LLIN. For example, in some areas the LLINs might have been distributed directly to each household, whereas in other areas, people will have gone to their local health centres to obtain their LLINs. There are also real or perceived differences in the availability of RDTs, effective anti-malarials and quality of care received at the health centres [19]. In Sunuhu, for example, the population is of a different ethnic origin than surrounding villages, more closely related to the population in Gwanga local level government (LLG) than Ilahita LLG. The Sunuhu population have less material wealth, less access to nutritious food, and rates of malnutrition and generally poor health are more common than in neighbouring villages. They may also be less likely to access care at the nearby health centre (due to distance, cultural difference, perceived benefit) and it is possible their access to LLINs has been reduced as a result.

Despite the decrease in prevalence and significant geographic heterogeneity, the genetic diversity of both *P. falciparum* and *P. vivax* appears to have been maintained at relatively high levels. Multiplicity of infection and especially the proportion of multiple clone infections has decreased. The proportion of multiple clone infections correlated well with prevalence and might be a suitable indicator of hotspot areas and areas of high transmission. Further investigation of markers that are not under selective pressure is required for a more detailed analysis of the impact of malaria control on genetic diversity, differentiation and population structure in this area [57, 58].

Although the prevalence of malaria has decreased across all age groups and there is no marked shift in the peak age of infections (compared to the 2005 survey and others [5]), there is a relatively higher proportion of symptomatic infections in the 2012/13 survey). This, together with a five- to ninefold increase in the proportion of symptomatic infections in adults is an indication that there might be reduced or delayed acquisition of immunity. More detailed investigations on the effect of the decreased transmission intensity on the incidence (and complexity) of malaria infections and clinical malaria episodes, and age/exposure related acquisition of clinical immunity are being conducted in several longitudinal child cohorts in East Sepik and Madang Province.

The impact on the prevalence of *P. vivax* was similar or higher as on *P. falciparum,* in 10 of 12 villages surveyed in both years. Based on the biology of relapsing *P. vivax* infections from hypnozoites and the fact that neither LLINs nor ACTs act to prevent these relapsing infections it is generally thought that *P. vivax* burden is more resilient to these tools and that an equivalent impact may not be observed in the same timeframe as for *P. falciparum*. In the same area as this study, a series of cohort studies showed that while *P. vivax* clinical episodes declined at rates comparable to *P. falciparum*, force of blood stage infections and prevalence took longer to decline [59]. The data presented here suggest that 8 years post-scale-up of LLINs appears to be a sufficient length of time for the hypnozoite burden to have been exhausted in most villages.

Although the two studies were not conducted at the same time of the year (April–May in 2005 vs. Oct-Feb in 2012–2013), this difference in timing is unlikely to substantially contribute to the observed decrease in prevalence between surveys. The ‘wet’ season is usually from October–April, whereas the so-called ‘dry’ season runs from May–September, however, in most parts of the lowlands of PNG there is perennial malaria transmission, with limited seasonality [2, 4, 8]. Previous repeated cross-sectional surveys in the neighbouring Wosera area showed no clear-cut seasonal patterns [3, 5] and a detailed paediatric longitudinal cohort showed increased transmission during the rainy season [60, 61]. In addition, a substantial decline in infection prevalence and clinical malaria episodes was also observed in three longitudinal child cohort studies conducted in the same area spanning a similar time period [59].

A limitation of the study was that the MM used to detect infections in the two time-periods was not the same. However, a previous study directly comparing qPCR and LDR-FMA reported substantial agreement between the two methods [32]. While the LDR-FMA detected slightly higher numbers of *P. falciparum* infections in that study (47% *vs* 41%) [32], this is not sufficient to be responsible for the observed difference in prevalence between the two studies, which mirrors the drop in prevalence by LM. The decreased prevalence of sub-microscopic *P. falciparum* and *P. vivax* infections in the 2012/13 study is potentially influenced by the difference between qPCR and LDR-FMA and thus the data from this earlier survey will appear to have a slightly higher proportion of sub-microscopic infections than when qPCR was used. In addition, 2nd reads of microscopy slides in 2012–2013 were performed based on qPCR results, potentially resulting in a lower proportion of sub-microscopic infection.

This study was not a formal component of the monitoring and evaluation of the national malaria control programme, with the primary aim to delve into the impact that reduced transmission is having on the epidemiology of malaria rather than assess the programme itself. Prevalence reported in this study is much higher than provincial averages from the national reports [22, 62], which to a large extent can be explained by the higher sensitivity of the MM that were used in these studies as compared to microscopy and RDTs. Molecular tools are much more sensitive at detecting low levels of parasitaemia and are therefore crucial to get a detailed insight on the prevalence of not only clinical disease, but also asymptomatic reservoirs of infection. Microscopic infections in the household, as well as gametocyte carriers were associated with high rate infection households in both years and in 2012–2013 many high rate households contained clinical cases, highlighting the utility of clinical and microscopy-based surveillance to identify transmission foci that could be specifically targeted with interventions aimed at reducing not only clinical cases, but the asymptomatic reservoir as well. Although the national programme is very effective in determining the impact on a national level, this study facilitates the investigation at a different level of sensitivity, geographical scale and subsequent detection of fine-scale heterogeneity in transmission.

Identifying and targeting focal points and hotspots of malaria is highly relevant for malaria control, since these are likely to be the areas where residual malaria transmission will persist, and can become an obstacle in efforts to eliminate malaria [28, 63, 64]. In addition they can play a catalysing role: hotspots fuel transmission within transmission foci, and interventions targeted at transmission hotspots therefore have the potential to reduce community-wide malaria transmission [28, 64]. Many of the high burden villages found in 2012/13 were also areas of the highest prevalence in 2005, and it remains to be seen if these same areas are still high in prevalence in future. If these hot spots are consistently at the same location, implementation of control tools to target them will be much easier. In other regions in the world, it has been observed that hotspots are remarkably stable even when transmission intensity declines, although clinical incidence might vary with time [28, 65–67]. In addition to scaling up conventional vector control tools such as LLINs and indoor residual spraying in these hotspot areas, other tools could be implemented that might prove more useful in these particular areas. Ongoing entomological studies in East Sepik and Madang provinces may advise on the suitability of additional vector control tools. Alternatively targeting the parasite via interventions to reduce the infectious reservoir in these communities, such as mass screening and treatment (MSAT) and mass drug administration (MDA). Modelling has shown that MDA targeting blood and liver stage drugs might be more effective in reducing *P. falciparum* and *P. vivax* prevalence than MSAT [68] and implementation of such strategies may be feasible to achieve in these relatively small communities.

## Conclusions

Although Papua New Guinea’s strengthened malaria control programme successfully reduced malaria prevalence nationwide between 2005 and 2013, with a substantial impact in ESP, areas with high ongoing transmission remain. In this study, LLIN coverage and use, socioeconomic factors, vector density/behaviour and environmental factors are the likely drivers of this heterogeneity and resilience in the face of control. In order to further reduce transmission in these areas, additional surveillance approaches, novel tools and community engagement strategies may need to be layered on top of sustained nationwide LLIN coverage and effective case management.

## Supplementary information

**Additional file 1.** Definition of anaemia. Definitions of anaemia that were used in the analyses stratified by age and sex. Values represent haemoglobin levels in g/L. Adapted from [35].

**Additional file 2.** Detailed demographic and clinical characteristics of study participants. Additional participants characteristics to Table 1.

**Additional file 3.** Allele frequencies of *Pfmsp1*, *Pvmsp1f3* and PvMS2 alleles in both surveys. Allele frequencies A) *Pfmsp1* alleles in 2005 (top) vs 2012/13 (bottom) of all alleles (left) and split into 3D7 (centre) and FC27 (right) allele families, and B) *Pvmsp1f3* and PvMS2 in 2005 (top) vs 2012/13 (bottom).

## Data Availability

The datasets generated during and/or analysed during the current study are not publicly available because it would compromise participant confidentiality and violates the ethical agreement in the informed consent forms. Data are however available upon reasonable request by contacting the PNG Medical Research Advisory Committee and the PNG Institute of Medical Research IRB. Please contact the corresponding author if you wish to access the data.

## Abbreviations

ACT: Artemisinin combination therapy
AIC: Akaike information criterion
aOR: Adjusted odds ratio
CI: 95% confidence interval
ESP: East Sepik Province, PNG
*He*: Expected heterogeneity
ITN: Insecticide treated net
LDR-FMA: Ligase detection reaction/microsphere assay
LLIN: Long-lasting insecticide net
LLG: Local level government
LM: Light microscopy
MDA: Mass drug administration
MFI: Median fluorescent intensity (from LDR-FMA)
MOI: Multiplicity of infection
MM: Molecular method, i.e. qPCR and/or LDR-FMA
MSAT: Mass screening and treatment
NMCP: PNG national malaria control programme
*Pfmsp2*: *P. falciparum* merozoite surface protein 2
PCR: Polymerase chain reaction
PNG: Papua New Guinea
PvMS2: *P. vivax* microsatellite marker 2
*Pvmsp1f3*: *P. vivax* merozoite surface protein 1–*f3* region
QMAL: Generic Pan *Plasmodium* PCR
qPCR: Quantitative PCR (real time PCR), in particular here this means the 18S rRNA detecting species specific qPCR
qRT-PCR: Reverse transcriptase PCR, in particular here this means the Pfs25 and Pvs25 qRT-PCR for gametocyte detection
RDT: Rapid diagnostic test
WBC: White blood cell
