# Supplementary material for: The epidemiology of Plasmodium falciparum and Plasmodium vivax in East Sepik Province, Papua New Guinea, pre- and post-implementation of national malaria control efforts
Source: Malar J. 2020 Jun 5;19:198. doi: 10.1186/s12936-020-03265-x (PMC7275396; doi:10.1186/s12936-020-03265-x)
Supplement: Supplementary file 1 — Additional file 1. Definition of anaemia. Definitions of anaemia that were used in the analyses stratified by age and sex. Values represent haemoglobin levels in g/L. Adapted from [35]. [file 12936_2020_3265_MOESM1_ESM.docx]

**Additional file 1**

Title: Definition of anaemia

Description: Definitions of anaemia that were used in the analyses stratified by age and sex. Values represent haemoglobin levels in g/L.

Adapted from: (35) WHO. Haemoglobin concentrations for the diagnosis of anaemia and assessment of severity. Vitamin and Mineral Nutrition Information System. Geneva; 2011.

| **Population** | **Non-anaemia** | **Mild anaemia** | **Moderate anaemia** | **Severe anaemia** |
| --- | --- | --- | --- | --- |
| **Children 6-59 months** | **≥110 g/L** | **100-109 g/L** | **70-99 g/L** | **<70 g/L** |
| **Children 5-11 years** | **≥115 g/L** | **110-114 g/L** | **80-109 g/L** | **<80 g/L** |
| **Children 12-14 years** | **≥120 g/L** | **110-119 g/L** | **80-109 g/L** | **<80 g/L** |
| **Non-pregnant women (≥15 years)** | **≥120 g/L** | **110-119 g/L** | **80-109 g/L** | **<80 g/L** |
| **Men (≥15 years)** | **≥130 g/L** | **110-129 g/L** | **80-109 g/L** | **<80 g/L** |
