# Supplementary material for: The epidemiology of Plasmodium falciparum and Plasmodium vivax in East Sepik Province, Papua New Guinea, pre- and post-implementation of national malaria control efforts
Source: Malar J. 2020 Jun 5;19:198. doi: 10.1186/s12936-020-03265-x (PMC7275396; doi:10.1186/s12936-020-03265-x)
Supplement: Supplementary file 2 — Additional file 2. Detailed demographic and clinical characteristics of study participants. Additional participants characteristics to Table 1. [file 12936_2020_3265_MOESM2_ESM.docx]

**Additional file 2**

Title: Detailed demographic and clinical characteristics of study participants.

Description: Additional participants’ characteristics to table 1

|  | **2005** | |  | **2013** | |  |
| --- | --- | --- | --- | --- | --- | --- |
|  | **n** | **%** |  | **n** | **%** | *p* |
| **Number of participants** | 2527 |  |  | 2486 |  |  |
| **Age group** |  |  |  |  |  | <0.001 |
| 0 to 3 years | 182 | 7.2% |  | 50 | 2.0% |  |
| >3 to 6 years | 214 | 8.5% |  | 267 | 10.7% |  |
| >6 to 9 years | 321 | 12.7% |  | 262 | 10.5% |  |
| >9 to 12 years | 265 | 10.5% |  | 226 | 9.1% |  |
| >12 to 20 years | 390 | 15.4% |  | 375 | 15.1% |  |
| >20 years | 1155 | 45.7% |  | 1306 | 52.5% |  |
| **Sex** |  |  |  |  |  | <0.001 |
| male | 1196 | 47.3% |  | 1048 | 42.2% |  |
| female | 1331 | 52.7% |  | 1438 | 57.8% |  |
| **Village** |  |  |  |  |  | <0.001 |
| 1. Bonohi | 174 | 6.9% |  |  |  |  |
| 2. Waragom | 175 | 6.9% |  | 95 | 3.8% |  |
| 3. Salata |  |  |  | 228 | 9.2% |  |
| 4. Urita | 166 | 6.6% |  | 177 | 7.1% |  |
| 5. Ilahita 3 | 148 | 5.9% |  | 146 | 5.9% |  |
| 6. Ilahita 4 | 175 | 6.9% |  | 165 | 6.6% |  |
| 7. Sunuhu | 188 | 7.4% |  | 189 | 7.6% |  |
| 8. Wapin | 145 | 5.7% |  |  |  |  |
| 9. Bangeleko | 172 | 6.8% |  |  |  |  |
| 10. Wapindumaka |  |  |  | 187 | 7.5% |  |
| 11. Wombisa | 174 | 6.9% |  | 212 | 8.5% |  |
| 12. Jama | 203 | 8.0% |  | 131 | 5.3% |  |
| 13. Sengo | 153 | 6.1% |  | 295 | 11.9% |  |
| 14. Maiwi | 133 | 5.3% |  | 70 | 2.8% |  |
| 15. Malba 1 | 163 | 6.5% |  | 250 | 10.1% |  |
| 16. Malba 2 | 174 | 6.9% |  | 179 | 7.2% |  |
| 17. Yenigo | 184 | 7.3% |  | 162 | 6.5% |  |
| **Reported use of bednets** | 2231 | 88.3% |  | 2348 | 94.91% | <0.001 |
| **History of recent malaria infection** | 409 | 16.3% |  | 137 | 5.5% | <0.001 |
| **History of recent malaria treatment** | 195 | 7.99% |  | 145 | 5.87% | 0.003 |
| **Anaemia** | 2131 | 85.7% |  | 1719 | 72.2% | <0.001 |
| Mild | 746 | 30.0% |  | 744 | 31.2% |  |
| Moderate | 1272 | 51.2% |  | 918 | 38.5% |  |
| Severe | 113 | 4.6% |  | 57 | 2.4% |  |
| No | 355 | 14.3% |  | 663 | 27.8% |  |
| **Current or recent reported febrile illness** | 186 | 7.4% |  | 356 | 14.4% | <0.001 |
| **Clinical Malaria** |  |  |  |  |  |  |
| *P. falciparum* and/or *P. vivax* | 92 | 3.6% |  | 58 | 2.3% | 0.007 |
| *P. falciparum* | 76 | 3.0% |  | 45 | 1.8% | 0.006 |
| P. vivax | 24 | 1.0% |  | 16 | 0.6% | 0.267 |
| Mixed *P. falciparum* and *P. vivax* | 8 | 0.3% |  | 3 | 0.12% | 0.226 |
