# Supplementary figures and images for: The epidemiology of Plasmodium falciparum and Plasmodium vivax in East Sepik Province, Papua New Guinea, pre- and post-implementation of national malaria control efforts
Source: Malar J. 2020 Jun 5;19:198. doi: 10.1186/s12936-020-03265-x (PMC7275396; doi:10.1186/s12936-020-03265-x)

A. *P. falciparum*

PfMSP2

2005

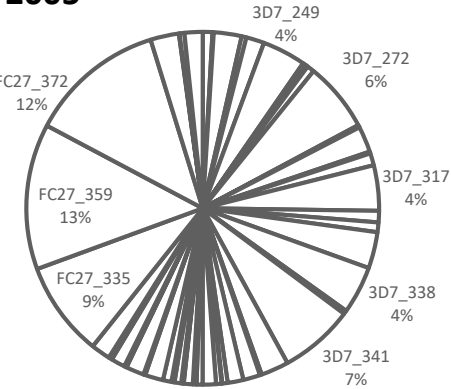

He = 0.94

3D7

FC27

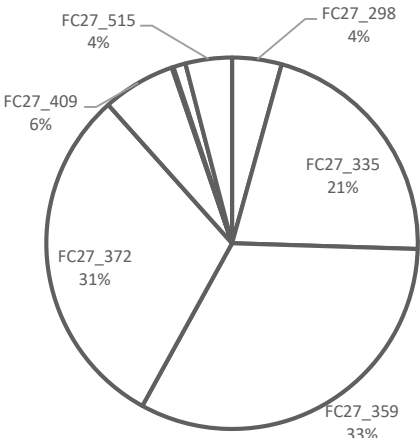

2012/13

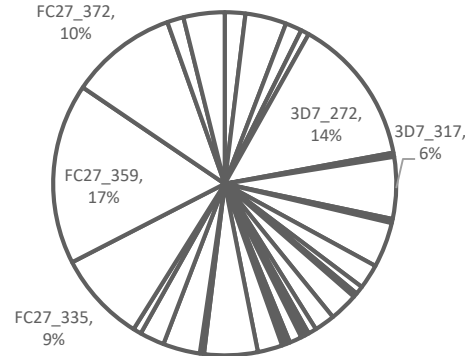

He = 0.92

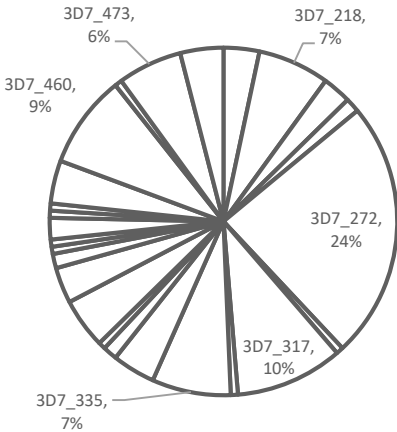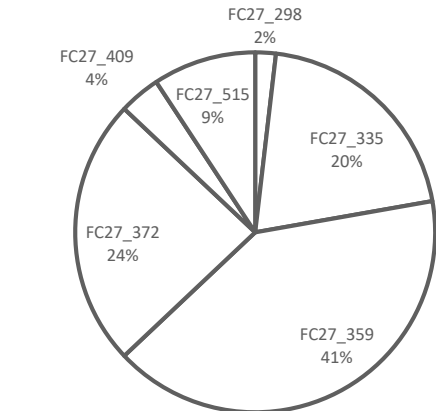

B. *P. vivax*

PvMSP1F3

MS2

2005

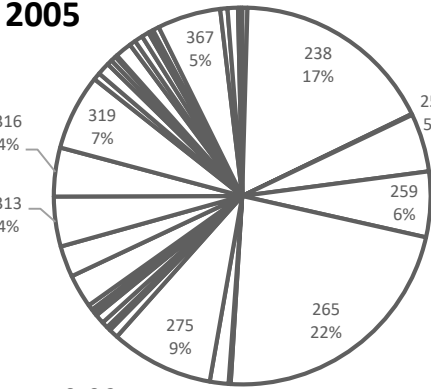

He = 0.92

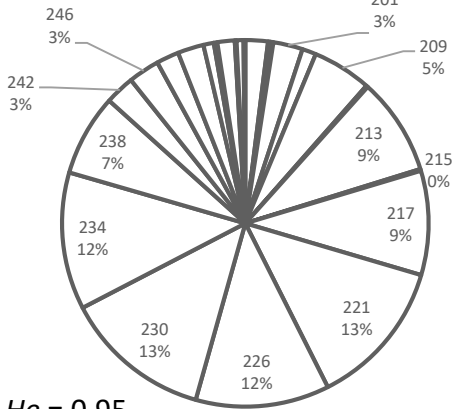

He = 0.95

2012/13

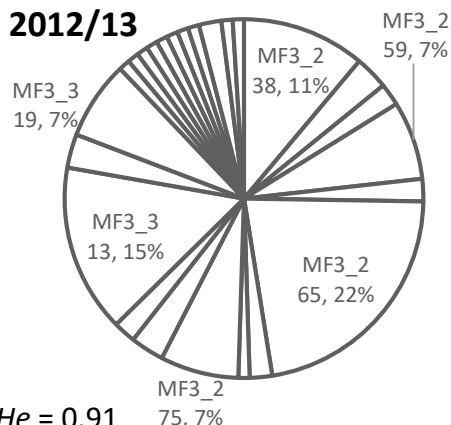

He = 0.91

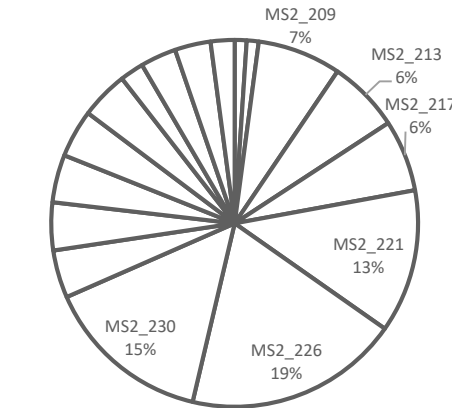

He = 0.91

Supplement: Supplementary file 3 — Additional file 3. Allele frequencies of Pfmsp1, Pvmsp1f3 and PvMS2 alleles in both surveys. Allele frequencies A) Pfmsp1 alleles in 2005 (top) vs 2012/13 (bottom) of all alleles (left) and split into 3D7 (centre) and FC27 (right) allele families, and B) Pvmsp1f3 and PvMS2 in 2005 (top) vs 2012/13 (bottom). [file 12936_2020_3265_MOESM3_ESM.pdf]
